# Renin as a Biomarker of Acute Kidney Injury and Mortality in Children With Severe Malaria or Sickle Cell Disease

**DOI:** 10.7759/cureus.45124

**Published:** 2023-09-12

**Authors:** Daniel Adan Jr., Anthony Batte, Ruth Namazzi, Ivan Mufumba, Caroline Kazinga, Kagan A Mellencamp, Caitlin Bond, Robert O Opoka, Chandy C John, Andrea L Conroy

**Affiliations:** 1 Pediatric Infectious Diseases and Global Health, Indiana University School of Medicine, Indianapolis, USA; 2 Child Health and Development Centre (CHDC), Makerere University College of Health Sciences, Kampala, UGA; 3 Paediatrics and Child Health, Makerere University College of Health Sciences, Kampala, UGA; 4 CHILD Laboratory, Global Health Uganda, Kampala, UGA; 5 Undergraduate Medical Education, Aga Khan University Medical College, Nairobi, KEN; 6 Center for Global Health, Indiana University School of Medicine, Indianapolis, USA

**Keywords:** mortality, acute kidney injury, severe malaria, sickle cell disease, renin

## Abstract

Background: Globally, a very high percentage of acute kidney injury (AKI) occurs in low- and middle-income countries (LMICs) where late recognition contributes to increased mortality. There are challenges with using existing biomarkers of AKI in LMICs. Emerging evidence suggests renin may serve as a biomarker of kidney injury that can overcome limitations in creatinine-based diagnostics.

Methods: Two study populations in Uganda were assessed. Cohort #1 was a two-site, prospective cohort study enrolling 600 children with severe malaria (SM). Cohort #2 was a prospective cohort study enrolling 185 children with sickle cell disease (SCD) hospitalized with a vaso-occlusive crisis. Plasma or serum renin concentrations were measured in both cohorts of children at the time of hospital admission using Luminex® (Luminex Corporation, Austin, Texas, United States) or enzyme-linked immunosorbent assay (ELISA), respectively. We assessed the ability of renin to discriminate between children with or without AKI and between children who survived and children who died using receiver operating characteristic curves.

Results: In both cohorts, renin concentrations were strongly associated with AKI and mortality. Renin was able to discriminate between children with or without AKI with an area under the curve (AUC) of 0.70 (95%CI, 0.65-0.74) in children with SM and 0.72 (95%CI, 0.6co3-0.81) in children with SCD. Renin was able to discriminate between children who survived and children who died with an AUC of 0.73 (95%CI, 0.63-0.83) in children with SM and 0.94 (95%CI, 0.89-0.99) in children with SCD. In Cohort #2, we compared renin against urine neutrophil gelatinase-associated lipocalin (NGAL) as the leading biomarker of AKI, and it had comparable performance in discriminating AKI and predicting mortality.

Conclusions: In two independent populations of children at risk of AKI with key differences in the etiology of kidney injury, renin was strongly associated with AKI and mortality and had moderate to good diagnostic performance to predict mortality.

## Introduction

Acute kidney injury (AKI) is increasingly recognized as an important clinical complication in hospitalized children in low- and middle-income countries (LMICs) and is associated with a substantial increase in morbidity and mortality [1,2]. AKI is an abrupt loss of kidney function and is defined based on an increase in serum creatinine or a decrease in urine output according to the Kidney Disease: Improving Global Outcomes (KDIGO) criteria [3]. Both diagnostic features can be challenging to assess in LMICs as creatinine testing is not always available due to limitations related to laboratory infrastructure, and limited nursing support makes assessment of urine output challenging.

Diagnosis of AKI using creatinine is based on a documented change in creatinine within a 48-hour to seven-day period from baseline. However, pre-illness creatinine values are unavailable for most children hospitalized with an acute illness, which necessitates an estimation of baseline creatinine. In addition, creatinine correlates with muscle mass and is impacted by the nutritional status of the population, which further complicates the estimation of normal creatinine in populations where malnutrition is common. While we have validated approaches to estimate baseline creatinine in African children [4], they rely on the assumption of normal kidney function before illness and may misclassify AKI in the context of pre-existing kidney disease and severe malnutrition. Thus, there is a desire to identify a kidney-specific biomarker that is independent of age and nutritional status to facilitate early identification of AKI.

AKI is a heterogeneous clinical condition characterized by multiple clinical phenotypes and mechanisms of injury [5]. The renin-angiotensin-aldosterone system (RAAS) is involved in regulating blood pressure, glomerular filtration rate (GFR), and fluid homeostasis through vasoconstriction. Renin has been identified as a biomarker of major adverse kidney events, AKI in cardiac surgery and sepsis, and impaired tissue perfusion in critically ill adults with septic shock [6-8]. However, there are limited data on renin in pediatric populations, particularly in LMICs where the burden of AKI is highest.

Due to the primary production of renin by the kidney, we sought to evaluate renin as a biomarker of AKI and mortality in hospitalized children. Specifically, by leveraging differences in study design and populations, we validated renin as a biomarker of AKI and mortality using independent cohorts of hospitalized children with key differences in AKI etiology and normal kidney function who were at risk of AKI.

## Materials and methods

Study design

In the present study, we evaluate the performance of serum or plasma renin as a biomarker of AKI (primary outcome) and mortality during the index hospitalization (secondary outcome) in two independent cohorts of Ugandan children hospitalized with acute severe malaria (SM) infection (Cohort #1) or pain crisis associated with sickle cell disease (SCD) (Cohort #2). Using data from a population of critically ill children with sepsis [9], we estimated that a minimum sample size of 141 was needed to detect a mean difference in renin of 2000 pg/mL in our primary outcome of AKI assuming an AKI prevalence of 35% from Cohort #2, a pooled standard deviation of 4000 pg/mL with 80% power at an alpha of 0.05. Based on the mortality rates in our respective cohorts, we would have 88% power to detect a minimum mean difference of 2000 pg/mL in Cohort #1 and 85% power to detect a mean difference in renin of at least 5000 pg/mL in Cohort #2 assuming a pooled standard deviation of 4000 pg/mL in both studies.

Study populations

Cohort #1

Between 2014 and 2017, 600 children hospitalized with SM aged six months to four years were enrolled from two referral hospitals in Central and Eastern Uganda: Mulago National Referral Hospital in Kampala, Central Uganda, and Jinja Regional Referral Hospital in Eastern Uganda [10,11]. Children were eligible for enrollment if they had diagnostic evidence of malaria with either direct visualization of parasites by Giemsa microscopy and features of SM (severe anemia, prostration, respiratory distress, multiple seizures, or coma) or a positive rapid diagnostic test for *Plasmodium falciparum* histidine-rich protein-2 (HRP-2). A history of chronic illness requiring medical care (including chronic kidney disease or known HIV infection), history of head trauma, coma, known developmental delay, cerebral palsy, or prior hospitalization for malnutrition were all exclusion criteria for children with SM. Delayed exclusion criteria included evidence of meningitis in children with decreased consciousness who had a lumbar puncture performed (white blood cell count > five cells/µL).

Cohort #2

Between January and August 2019, 185 children with SCD hospitalized with a vaso-occlusive crisis were consecutively enrolled at Mulago National Referral and Teaching Hospital in Central Uganda [12,13]. Children were eligible if they had documented SCD by hemoglobin electrophoresis (HbSS), were aged 2-18 years, had pain score ≥ two on an age-specific pain scale, and were willing to complete the study procedures. Pain was assessed using the face, legs, activity, cry, and consolability (FLACC) scale in children aged two to three years, the Wong-Baker Faces pain scale in children aged three to seven years, and the numeric pain scale in children greater than eight years of age. There were no exclusion criteria for comorbid conditions, including pre-existing kidney disease in this population.

Study procedures

At enrollment, all children had a physical exam and a complete history performed as well as a blood draw for clinical and study-related procedures. Whole blood was collected in additive-free tubes and ethylenediamine tetraacetic acid (EDTA)-anticoagulated BD Vacutainer® blood collection tubes (Becton, Dickinson and Company, Franklin Lakes, New Jersey, United States) for isolation of plasma and serum, respectively. A complete blood count was performed on EDTA-anticoagulated blood on admission. Peripheral blood smears were used to quantify parasite density using Giemsa staining with standard protocols in both cohorts. In Cohort #2, the mean of three independent measurements was used to calculate blood pressure, and hypertension was defined as a systolic blood pressure > 95% or a diastolic blood pressure > 95% for children < 13 years of age using age- and sex-specific norms or a systolic blood pressure ≥ 130mmHg or diastolic blood pressure ≥ 80 for children 13 years of age or older [14]. For both cohorts, heights and weights were converted into z scores (height-for-age, weight-for-age, weight-for-height, or BMI-for-age) based on WHO growth references [12].

All children had blood collected at enrollment for malaria evaluation, a complete blood count, and a Point-of-Care i-STAT test using the CHEM8+ cartridge that measures metabolic status and renal function (Abbott Point of Care Inc., Princeton, New Jersey, United States) [12]. In Cohort #2, a spot urine sample was collected using a urine bag or urine container for older children and sent to the laboratory within two hours of collection for urinalysis and urine neutrophil gelatinase-associated lipocalin (uNGAL) dipstick as a structural biomarker of AKI [12]. Urine samples were spun at room temperature for five minutes at 400g and collected and stored at -80°C until testing. Levels of uNGAL were tested in batches on stored samples by enzyme-linked immunosorbent assay (ELISA), according to the manufacturer’s protocol (Kit 036; BioPorto Diagnostics Inc., Denmark, South Carolina, United States) as described [12]. Urine samples were diluted 1:1000, and the lower and upper limits of the assay were five and 2000 ng/mL, respectively [12]. All technicians who conducted the testing were blinded to participant details.

Defining AKI

AKI was defined using the KDIGO criteria based on a 1.5-fold increase in creatinine over the estimated baseline or a 0.3mg/dL change in creatinine within 48 hours in both cohorts [3,15]. Staging was as follows: Stage one, 1.5-1.9-fold increase in creatinine over baseline; Stage two, 2.0-2.9-fold increase over baseline; Stage three, ≥ 3.0-fold increase over baseline [3]. For children with SM, baseline creatinine was estimated using a height-independent approach to back-calculate creatinine assuming a normal GFR of 120mL/min per 1.73m^2^ as described [3,4,16,17]. AKI was defined using creatinine measured in a reference laboratory using the modified Jaffe method, which is isotope dilution mass spectrometry (IDMS)-traceable. In children with SCD in Cohort #2, creatinine was measured at enrollment, at 48 hours, and at day seven or at discharge by iSTAT using an enzymatic assay traceable to the United States National Institute of Standards and Technology standard reference material SRM909, with a reportable range of 0.20-20.0mg/dL [12]. Creatinine values that were below the reportable range were assigned a value of 0.19mg/dL. The lowest measured creatinine value was taken as the participants' baseline. In instances where only a single creatinine measure was available (n=7), the Pottel age-based GFR estimating equation [18] was used to back-calculate baseline creatinine, assuming a normal GFR of 120mL/min per 1.73m^2^ [4,12].

Assessment of renin

In Cohort #1, renin was assessed on admission plasma samples stored at -80˚C using a Luminex® MAGPIX® bead-based immunoassay with a four-fold dilution (R&D Systems, Inc., Minneapolis, Minnesota, United States) and a range of detection of eight pg/mL to 324,480 pg/mL and a mean coefficient of variation of 4.2%. In Cohort #2, renin was assessed on admission serum samples stored at -80˚C by ELISA using a five-fold dilution (DuoSet® ELISA Development Systems, R&D Systems, Inc.) with a range of detection of 156 pg/mL to 40,000 pg/mL and a mean coefficient of variation of 8.3%.

Statistical analysis

Data were analyzed using Stata Statistical Software: Release 17 (2021; StataCorp LLC, College Station, Texas, United States) and GraphPad Prism v9.1 (2021; Dotmatics, Boston, Massachusetts, United States). Data are presented descriptively using the median (interquartile range (IQR)) or mean (standard deviation (SD)). Categorical variables are presented as the number and frequency and differences in groups compared using Pearson’s chi-squared test. Differences in median renin levels by AKI and mortality status were assessed using the Wilcoxon rank-sum test. Linear regression was used to assess the relationship between age, nutritional status, and renin levels. To assess the discriminatory ability of renin, non-parametric receiver operating characteristic (ROC) curve analysis was performed in Cohort #1 and validated in Cohort #2. To compare the areas under independent ROC curves (AUC), we used the method of Delong et al. [19].

Ethical considerations

For Cohort #1,approval was granted by the Institutional Review Boards at Makerere University School of Medicine (approval number: 2013-141), the University of Minnesota (approval number: 1309M42501), and Indiana University (approval number: 1412213778). The Uganda National Council for Science and Technology approved the study (approval number: HS1522).

For Cohort #2, approval was granted by the Makerere University School of Biomedical Sciences Research and Ethics Committee (approval number: SBS-S46) and the Uganda National Council for Science and Technology (approval number: HS 2443). All methods were carried out in accordance with relevant guidelines and regulations. Written informed consent was obtained from the parents or legal guardians of all study participants.

## Results

**Figure 1:**
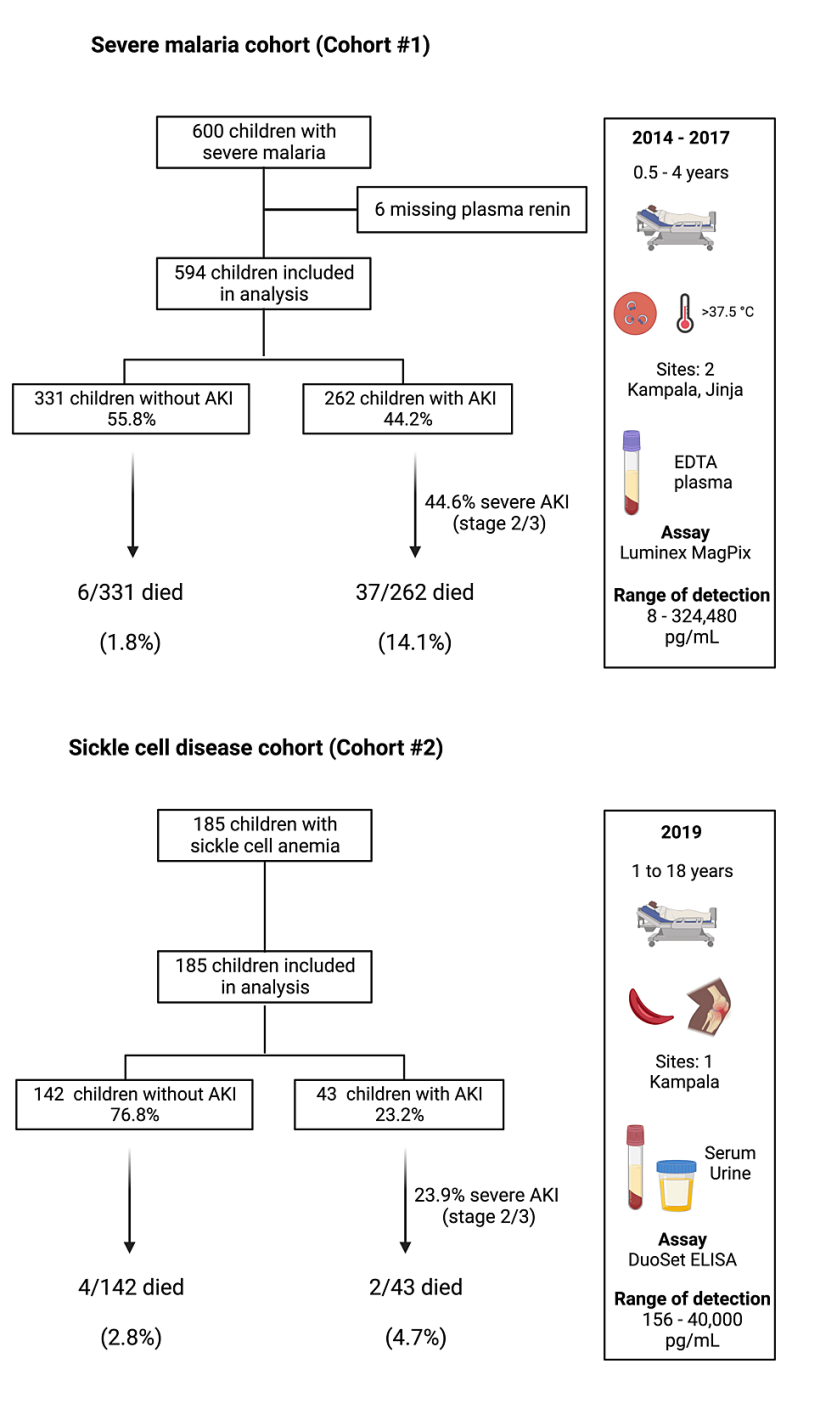
Study flow diagram for children with severe malaria and children with sickle cell disease. The frequency of AKI in both cohorts is depicted in the flow diagram. Cohort and demographic characteristics as well as testing procedures are illustrated in the chart. AKI: acute kidney injury; EDTA: ethylenediamine tetraacetic acid Manufacturer details: Luminex® MAGPIX®, R&D Systems, Inc., Minneapolis, Minnesota, United States; DuoSet® ELISA Development Systems, R&D Systems, Inc., Minneapolis, Minnesota, United States

We evaluated renin levels in 779 hospitalized children where 594 were hospitalized with SM (Cohort #1) (Figure 1, Table 1) and 185 were hospitalized with vaso-occlusive crisis (Cohort #2) (Figure 1, Table 1). Among children with SM, the prevalence of AKI on admission was 44.2%, and 7.3% of children died. Among children with SCD, the prevalence of AKI on admission was 23.2%, and 3.2% of children died. Children with SM had more severe AKI and signs of disease severity with a higher frequency of coma and respiratory distress, while children with SCD were more likely to have severe anemia and hyperfiltration. Consistent with known patterns of disease presentation, children with SM were younger than children with SCD (Table 1).

**Table 1:** Participant characteristics. Unless otherwise specified, data are presented as mean (SD) for demographic and admission characteristics and median (IQR) for laboratory characteristics. For measures with incomplete data, the number of observations is noted after the (SD) or (IQR). ^a^ Weight-for-age z scores available for children < 10 years; ^b^ Weight-for-height z scores available for children < 5 years; ^c^ BMI-for-age z scores available for children ≥ 5 years.

	Cohort #1: Severe malaria (N=594)	Cohort #2: Sickle cell disease (N=185)
Demographic and admission characteristics:		
Age in years	2.1 (0.92)	8.9 (4.0)
Sex (Female), n (%)	258 (43.4)	77 (41.6)
Site:		
Jinja, n (%)	266 (44.8)	-
Mulago, n (%)	328 (55.2)	185 (100)
Weight-for-age z score^a^	-1.1 (1.1), 587	-1.3 (1.2), 112
Height-for-age z score	-1.1 (1.3)	-1.4 (1.4), 184
Weight-for-height z score^b^	-0.67 (1.1), 587	-1.4 (1.2), 36
BMI-for-age z score^c^	-	-1.3 (1.5), 149
Temperature, ˚C	37.7 (1.2)	37.3 (0.86)
Respiratory rate, /min	50.6 (13.4)	31.6 (10.3)
Pulse, /min	158 (23.2)	110 (19.4)
Coma, n (%)	72 (12.1), 593	4 (2.2)
Respiratory distress, n (%)	175 (29.5)	32 (17.3)
Severe anemia, n (%)	303 (51.0)	131 (70.8)
Laboratory characteristics:		
Hemoglobin, g/dL	5.7 (3.6, 8.6), 591	7.2 (6.3, 8.2)
White blood cell, 10^3^/µL	12.1 (8.7, 19.2), 591	22.6 (16.7, 33.4)
Platelet count, 10^3^/µL	113 (62, 216), 591	418 (306, 525)
Creatinine, mg/dL	0.37 (0.29, 0.48), 593	0.3 (0.19, 0.4)
BUN, mg/dL	12 (7, 21), 591	4 (3, 7)
Sodium, mmol/L	135 (133, 138), 554	138 (135, 140)
Potassium, mmol/L	4.2 (3.8, 4.6), 553	3.8 (3.6, 4.1), 184
TCO_2_, mmol/L	15 (11, 18), 547	21 (19, 22)
Anion gap, mmol/L	21 (18, 23), 512	17 (16, 18), 183
Kidney function:		
AKI on admission, n (%)	262 (44.2), 593	43 (23.2)
Severe AKI: Stage 2+, n (%)	117 (19.7), 593	22 (11.9)
Hyperfiltration: eGFR > 185 mL/min per 1.73 m^2^, n	7 (1.2), 593	90 (48.9), 184
Outcome:		
In-hospital mortality, n (%)	43 (7.3)	6 (3.2)

Renin in SM (cohort #1)

**Figure 2:**
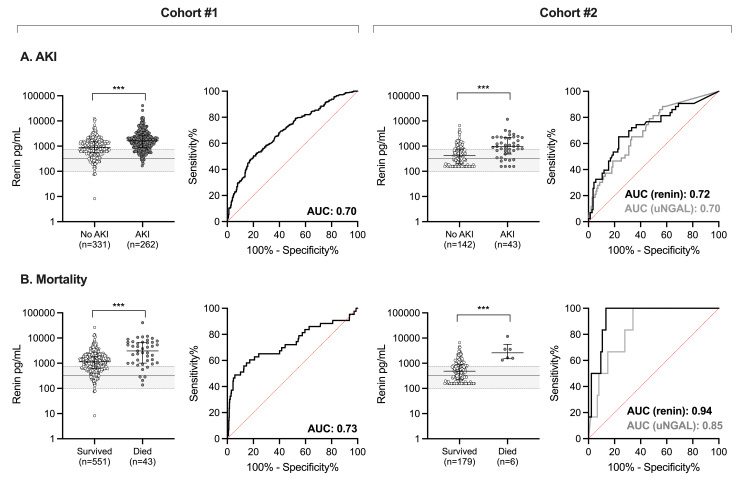
Ability of renin to predict AKI or in-hospital mortality in the two cohorts. (A) ROC curve showing the ability of increasing renin concentrations to predict AKI in children with SM (Cohort #1) or increasing renin and uNGAL concentrations to predict AKI in children with SCD (Cohort #2); (B) ROC curve showing the ability of increasing renin concentrations to predict mortality in children with SM (Cohort #1) or increasing renin and uNGAL concentrations to predict mortality in children with SCD (Cohort #2). Scatter plots depict renin concentrations with the median and IQR for children with SM or SCD. Gray shading on the plot represents the median (IQR) of renin levels measured in the community children from Cohort #1 as a population reference. Wilcoxon rank-sum test was used where ***p<0.001. The data are presented on log-transformed scale. ROC: receiver operating characteristic; AKI: acute kidney injury; SM: severe malaria; uNGAL: urine neutrophil gelatinase-associated lipocalin: SCD: sickle cell disease; IQR: interquartile range; AUC: area under the curve

To determine whether renin levels increased in the context of AKI, we compared median renin levels in children based on AKI status. Among children with SM, renin levels were higher in children with AKI on admission compared to children without AKI (median 1627 pg/mL (IQR 895, 2612) vs. 900 pg/mL (IQR 557, 1483), p<0.0001) (Figure 2A) and was associated with persistent AKI at the 24-hour follow-up (p<0.0001). Further, renin levels were higher in children who died compared to children who survived (median 3078 pg/mL (IQR 1011, 6755) vs. 1139 pg/mL (IQR 619, 1852), p<0.0001) (Figure 2B). To evaluate the potential utility of renin as a biomarker to discriminate between children with or without AKI or at increased risk of death, we used ROC curves. Renin had a moderate ability to discriminate between children with or without AKI with an area under the curve (AUC) of 0.70 (95% CI, 0.65-0.74) (Figure 2A). Renin also showed moderate ability to predict mortality with an AUC of 0.73 (95%CI, 0.63-0.83) (Figure 2B).

Renin in SCD

To validate these findings in an independent cohort of children with differences in AKI etiology, we measured renin concentrations in children with SCD hospitalized with an acute pain crisis. Consistent with findings in children with SM from Cohort #1, renin was elevated in children with SCD-associated AKI compared to children without AKI (median 955 pg/mL (IQR 486, 2185) vs. 420 pg/mL (IQR 197, 723), p<0.0001) (Figure 2A). Further, among children with SCD, renin levels were higher in children who died compared to those who survived (median 2605 pg/mL (IQR 1585, 3588) vs. median 486 pg/mL (IQR 222, 880), p=0.0003) (Figure 2B). By ROC curve analysis, renin showed moderate ability in discriminating AKI (AUC, 0.72; 95%CI, 0.63-0.81) (Figure 2A) and strong ability to predict mortality in children with SCD (AUC, 0.94; 95%CI, 0.89-0.99) (Figure 2B).

To evaluate renin’s performance against a standard AKI biomarker, we compared the AUC between renin and uNGAL. Among children who had both serum renin and uNGAL measured (n=182), renin was comparable to uNGAL as a biomarker of AKI with an AUC of 0.72 (95%CI, 0.63-0.81) compared to 0.70 (95%CI, 0.62-0.79), respectively (p=0.72). Furthermore, renin had better discriminatory ability when assessing mortality demonstrated with an AUC of 0.94 (95%CI, 0.88-0.99) in comparison to an AUC of 0.85 (95%CI, 0.74-0.96) for uNGAL (p=0.04). The performance of renin as a biomarker relative to uNGAL is presented in Table 2.

**Table 2:** Test characteristics of renin as a biomarker of AKI and mortality. Values presented as % (95%CI) except for AUROC. Optimal renin cutoffs derived from Youden Index for each outcome and cohort. AUROC: area under the receiver operating curve; AKI: acute kidney injury; NGAL: neutrophil gelatinase-associated lipocalin

Outcome	Cohort #1	Cohort #2	
	Renin (plasma)	Renin (serum)	NGAL (urine)
AKI on admission	Optimal cutoff = 1611 pg/mL	Optimal cutoff = 745 pg/mL	Optimal cutoff = 150 ng/mL
AUROC (95% CI)	0.70 (0.65-0.74)	0.72 (0.63-0.81)	0.70 (0.62-0.79)
Sensitivity	50 (44-57)	65 (49-79)	26 (14-41)
Specificity	80 (75-84)	77 (69-83)	93 (87-97)
Positive predictive value	66 (59-72)	46 (33-59)	52 (30-74)
Negative predictive value	67 (62-72)	88 (81-93)	80 (73-86)
In-hospital mortality	Optimal cutoff = 2342 pg/mL	Optimal cutoff = 1323 pg/mL	Optimal cutoff = 150 ng/mL
AUROC (95% CI)	0.73 (0.63-0.83)	0.94 (0.89-0.99)	0.85 (0.74-0.96)
Sensitivity	58 (42-73)	100 (54-100)	50 (12-88)
Specificity	85 (82-88)	87 (81-91)	90 (84-94)
Positive predictive value	24 (16-33)	20 (8-39)	14 (3-36)
Negative predictive value	96 (94-98)	100 (98-100)	98 (95-100)

Characteristics of renin as a biomarker

An ideal biomarker of AKI would not be impacted by characteristics such as age, sex, or nutritional status. To determine whether renin varied by age or nutritional status, we evaluated renin in a population of community children from the same household compound area or nearby communities from the children enrolled with SM from Cohort #1. Using linear regression, we assessed whether renin was associated with age or nutritional status. For a one-unit change in age, there was a -0.07 change in log_e_ renin levels (95%CI, -0.19-0.05; p=0.227). Similarly, for a one-unit change in weight-for-height z score, there was a 0.06 change in log_e_ renin levels (95%CI, -0.06-0.17; p=0.348). In contrast, creatinine levels increased with age (beta= 0.02, 95%CI, 0.01-0.03; p=0.004).

## Discussion

In this study, renin was assessed as a biomarker of AKI and mortality in a cohort of children with SM and a cohort of children with SCD hospitalized with an acute pain crisis. By leveraging two populations of Ugandan children that differ based on age, the presence of pre-existing kidney disease, and underlying mechanisms of AKI, we were able to evaluate and validate renin as a biomarker of AKI and mortality in children. Despite key differences in the etiology of AKI between these two cohorts, renin was consistently elevated in AKI and associated with mortality. Despite differences in the sample population and assays used, the differences in renin levels were consistent across both cohorts. Together, these data support renin as a biomarker of AKI associated with adverse clinical outcomes. Thus, use of renin as a biomarker may help identify children with increased clinical risk in need of further evaluation of kidney function and more advanced supportive care.

Renin has been identified as a biomarker of AKI and mortality in various patient populations mostly consisting of older adults. Among a population of older adults on cardiopulmonary bypass, a larger change in renin levels was associated with increased duration of vasopressor use, ICU and hospital stay, and was associated with cardiac instability and AKI [20]. Further, in a population of critically ill older adults, serum renin concentrations were independently associated with major adverse kidney events and mortality [7]. Studies evaluating renin alongside venous lactate as an alternative measure of impaired tissue perfusion suggest renin may be a complementary measure that can contribute to [6], or outperform [8], lactate. Recently, data were reported from a population of critically ill children with septic shock where renin and prorenin trends across 72 hours had moderate ability to predict severe persistent AKI and mortality [9].

Our study confirms and extends the findings from critically ill children hospitalized in a high-income setting and demonstrates that renin has moderate and comparable performance to discriminate AKI in two populations of children in Uganda using a single measure of renin on admission. Additional studies are needed to evaluate the utility of renin to identify AKI using AKI definitions that incorporate structural markers of kidney injury.

While studies in high-income settings have used multiple measures of renin to identify patients at risk of major adverse kidney events, mortality, or prolonged ICU stay, this may not be feasible in resource-limited settings. Hypovolemia and hypoperfusion due to inadequate oral fluid intake complicated by fever or gastrointestinal losses in children due to diarrhea and vomiting could lead to reduced renal perfusion and renin release [21]. Although multiple measures of renin may not be possible in lower-income settings, we could overcome these challenges by assessing renin after fluid resuscitation or stabilization. As renin was unaffected by age and nutritional status, it offers advantages over creatinine. Additional studies are needed to evaluate the utility of a single renin measure and evaluate its performance in combination with other functional or structural measures such as creatinine or NGAL, respectively. An AKI biomarker must be tailored to the population, resources, and setting of interest. Ultimately, biomarkers that can be adapted to a point-of-care test offer advantages in LMICs where limitations in laboratory testing contribute to late recognition of AKI.

Despite key differences in the two cohorts included in the present study, we observed consistent renin-angiotensin-aldosterone system (RAAS) dysfunction. AKI in severe malaria is attributed to a decrease in tissue perfusion related to hypovolemia, vascular dysfunction, and parasite sequestration in the microvasculature [21]. Additional mechanisms of injury include tubulointerstitial inflammation and injury and oxidative stress related to hemolysis [22-24]. SM often occurs in previously healthy children where the prevalence of preexisting kidney disease is expected to be low [4]. In contrast, children with SCD suffer from multiple renal complications with impaired urinary concentrating ability, distal nephron dysfunction, cortical hypoperfusion, and medullary hypoperfusion [25]. Early in life, children with SCD experience hyperfiltration followed by a progressive loss in filtration and chronic kidney disease onset in the second to third decade of life [25]. In the cohort of children with SCD, the frequency of hyperfiltration was 48.9% with an AKI prevalence on admission of 23.2%. In both cohorts of children, renin levels were comparable and had similar performance when discriminating between children with and without AKI. The ability of renin to discriminate between survivors and non-survivors was moderate in children with SM and good in children with SCD. Thus, renin seems to be a consistent biomarker of the RAAS pathway associated with AKI, adverse clinical outcomes in adults and children, and mortality.

Strengths and limitations

Strengths of the study included the use of two well-characterized cohorts of Ugandan children with AKI, which enabled us to assess the generalizability of the findings by leveraging the unique differences of the populations. While there were differences in the sample matrix (plasma vs. serum) and assay used (Luminex vs. ELISA), the results were consistent, which further supports renin as a biomarker of AKI and mortality.

Limitations to the studies included a single renin measure on enrollment, which precluded analyses to evaluate whether changes in renin following correction for hypovolemia would better predict AKI and mortality. Further, we were unable to correlate renin levels with objective measures of hemodynamic status at enrollment. An important consideration for this study is the inability to use a direct active renin assay that allows for comparison with existing literature on renin, and the renin levels measured in this study likely reflect both renin and prorenin which can be 10-fold higher than active renin and are less impacted by acute stimuli. Future work should focus on addressing this knowledge gap. Evaluation of renin as a biomarker of AKI is complicated by the fact that there are limitations to creatinine-based diagnostics. Future studies should assess renin in conjunction with urine output-based AKI definitions. To overcome limitations in creatinine-based diagnostics, we used mortality as the endpoint in this study. Further, we included uNGAL as an alternative AKI biomarker for comparison and demonstrated that renin is a good predictor of mortality.

## Conclusions

Renin has been identified as a biomarker in several different patient populations including sepsis, cardiovascular disease, and cardiac surgery, where renin was strongly associated with in-hospital mortality. In the present study, we validate these findings using two independent cohorts of children with AKI in a LMIC setting. As an estimated 85% of AKI occurs in LMICs, there is an urgent need for studies to evaluate potential AKI biomarkers globally where there is considerable variation in population genetic diversity and exposures leading to AKI.

## References

[1] Goldstein SL, Akcan-Arikan A, Alobaidi R (2022). Consensus-based recommendations on priority activities to address acute kidney injury in children: a modified Delphi consensus statement. JAMA Netw Open.

[2] Cerdá J, Mohan S, Garcia-Garcia G (2017). Acute kidney injury recognition in low- and middle-income countries. Kidney Int Rep.

[3] (2012). KDIGO clinical practice guideline for acute kidney injury. Kidney Int Suppl.

[4] Batte A, Starr MC, Schwaderer AL (2020). Methods to estimate baseline creatinine and define acute kidney injury in lean Ugandan children with severe malaria: a prospective cohort study. BMC Nephrol.

[5] Zarbock A, Nadim MK, Pickkers P (2023). Sepsis-associated acute kidney injury: consensus report of the 28th Acute Disease Quality Initiative workgroup. Nat Rev Nephrol.

[6] Leśnik P, Łysenko L, Krzystek-Korpacka M, Woźnica-Niesobska E, Mierzchała-Pasierb M, Janc J (2022). Renin as a marker of tissue perfusion, septic shock and mortality in septic patients: a prospective observational study. Int J Mol Sci.

[7] Flannery AH, Ortiz-Soriano V, Li X (2021). Serum renin and major adverse kidney events in critically ill patients: a multicenter prospective study. Crit Care.

[8] Gleeson PJ, Crippa IA, Mongkolpun W (2019). Renin as a marker of tissue-perfusion and prognosis in critically ill patients. Crit Care Med.

[9] Stanski NL, Pode Shakked N, Zhang B (2023). Serum renin and prorenin concentrations predict severe persistent acute kidney injury and mortality in pediatric septic shock. Pediatr Nephrol.

[10] Conroy AL, Opoka RO, Bangirana P (2019). Acute kidney injury is associated with impaired cognition and chronic kidney disease in a prospective cohort of children with severe malaria. BMC Med.

[11] Conroy AL, Hawkes M, Elphinstone RE (2016). Acute kidney injury is common in pediatric severe malaria and is associated with increased mortality. Open Forum Infect Dis.

[12] Batte A, Menon S, Ssenkusu JM (2022). Neutrophil gelatinase-associated lipocalin is elevated in children with acute kidney injury and sickle cell anemia, and predicts mortality. Kidney Int.

[13] Batte A, Menon S, Ssenkusu J (2022). Acute kidney injury in hospitalized children with sickle cell anemia. BMC Nephrol.

[14] Flynn JT, Kaelber DC, Baker-Smith CM (2017). Clinical practice guideline for screening and management of high blood pressure in children and adolescents. Pediatrics.

[15] Namazzi R, Batte A, Opoka RO (2022). Acute kidney injury, persistent kidney disease, and post-discharge morbidity and mortality in severe malaria in children: a prospective cohort study. EClinicalMedicine.

[16] Schwartz GJ, Muñoz A, Schneider MF, Mak RH, Kaskel F, Warady BA, Furth SL (2009). New equations to estimate GFR in children with CKD. J Am Soc Nephrol.

[17] Chawla LS, Bellomo R, Bihorac A (2017). Acute kidney disease and renal recovery: consensus report of the Acute Disease Quality Initiative (ADQI) 16 workgroup. Nat Rev Nephrol.

[18] Pottel H, Hoste L, Martens F (2012). A simple height-independent equation for estimating glomerular filtration rate in children. Pediatr Nephrol.

[19] DeLong ER, DeLong DM, Clarke-Pearson DL (1988). Comparing the areas under two or more correlated receiver operating characteristic curves: a nonparametric approach. Biometrics.

[20] Küllmar M, Saadat-Gilani K, Weiss R (2021). Kinetic changes of plasma renin concentrations predict acute kidney injury in cardiac surgery patients. Am J Respir Crit Care Med.

[21] Batte A, Berrens Z, Murphy K, Mufumba I, Sarangam ML, Hawkes MT, Conroy AL (2021). Malaria-associated acute kidney injury in African children: prevalence, pathophysiology, impact, and management challenges. Int J Nephrol Renovasc Dis.

[22] Elphinstone RE, Conroy AL, Hawkes M (2016). Alterations in systemic extracellular heme and hemopexin are associated with adverse clinical outcomes in Ugandan children with severe malaria. J Infect Dis.

[23] Plewes K, Kingston HW, Ghose A (2018). Acetaminophen as a renoprotective adjunctive treatment in patients with severe and moderately severe falciparum malaria: a randomized, controlled, open-label trial. Clin Infect Dis.

[24] Barber BE, Grigg MJ, Piera KA (2018). Intravascular haemolysis in severe Plasmodium knowlesi malaria: association with endothelial activation, microvascular dysfunction, and acute kidney injury. Emerg Microbes Infect.

[25] Nath KA, Hebbel RP (2015). Sickle cell disease: renal manifestations and mechanisms. Nat Rev Nephrol.

